# Cushing´s syndrome: high aggressiveness of low-grade tumors

**DOI:** 10.11604/pamj.2020.36.315.23901

**Published:** 2020-08-21

**Authors:** Faycal El Guendouz, Sara Derrou, Fouad Atoini, Hassan Ouleghzal, Somaya Safi

**Affiliations:** 1 Department of endocrinology, Moulay Ismail Military Hospital of Meknes, Meknes, Morocco,; 2 Sidi Mohamed Ben Abdellah University Fez, Fez Morocco,; 3 Department of thoracic surgery, Moulay Ismail Military Hospital of Meknes, Meknes, Morocco

**Keywords:** Cushing’s syndrome, exposition, supraphysiological, endogenous, exogenous, cortisol

## Abstract

Cushing´s syndrome is caused by prolonged exposition supraphysiological to endogenous or exogenous cortisol. Ectopic production of adrenocorticotropic hormone by lung carcinoid tumors is relatively rare. Most documented cases have been reported individually. This rare neoplasm low grade that may secrete adrenocorticotropin (ACTH) leading to rapid development of hypercortisolism which is the main mode of discovery, can be a real aggressive form. This report shows a high aggressiveness of this endocrine neoplasia, wich was marked on the general, morphological, bone and psychiatric level. The trivialization of clinical signs had caused the delay in diagnosis with irreparable bone consequences.

## Introduction

Cushing´s syndrome (CS) is caused by prolonged exposition supraphysiological to endogenous or exogenous cortisol. Ectopic production of adrenocorticotropic hormone by carcinoid tumors is relatively rare and most documented cases have been reported individually [1]. This rare neoplasm low grade that may secrete adrenocorticotropin (ACTH) leading to rapid development of hypercortisolism which is the main mode of discovery, can be a real aggressive form. This report shows a high aggressiveness of this endocrine neoplasia, wich was marked on the general, morphological, bone and psychiatric level. A complete response was objectified after surgery.

## Patient and observation

**Figure 1:**
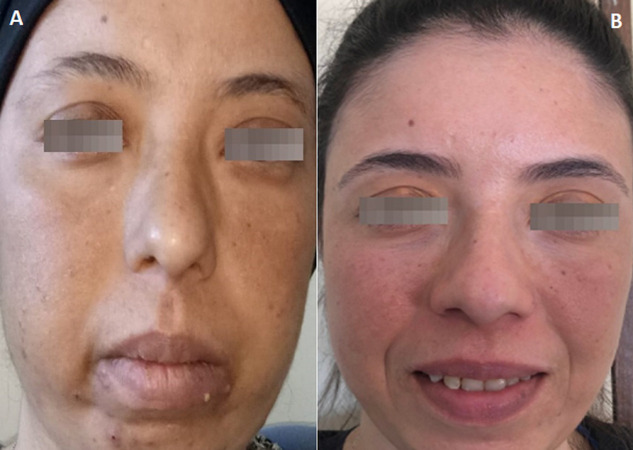
face picture before (A) and one year after surgery (B)

**Figure 2:**
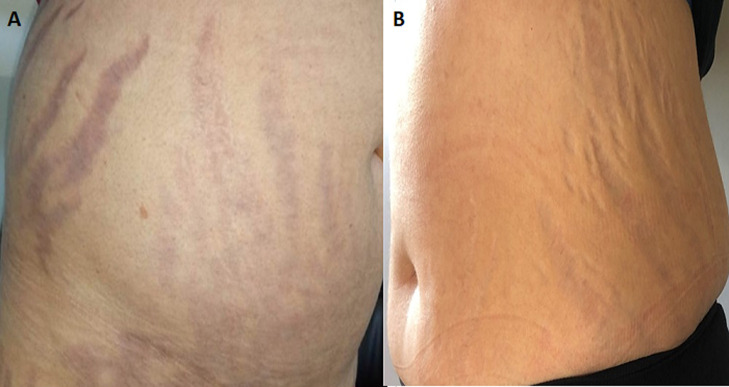
flanks pictures before (A) and one year after surgery (B), showing purple striae

**Figure 3:**
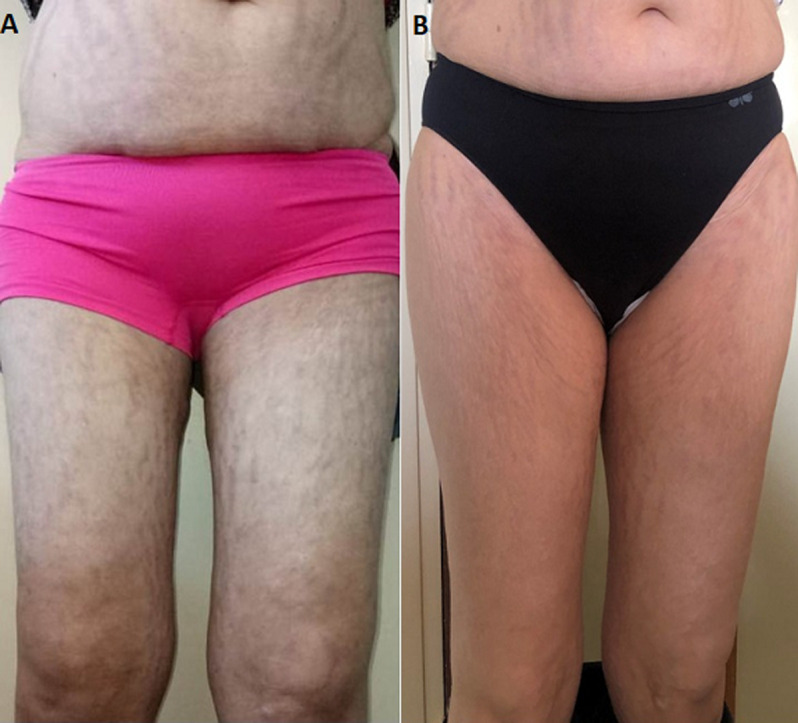
thighs pictures before (A) and one year after surgery (B), showing purple striae

**Figure 4:**
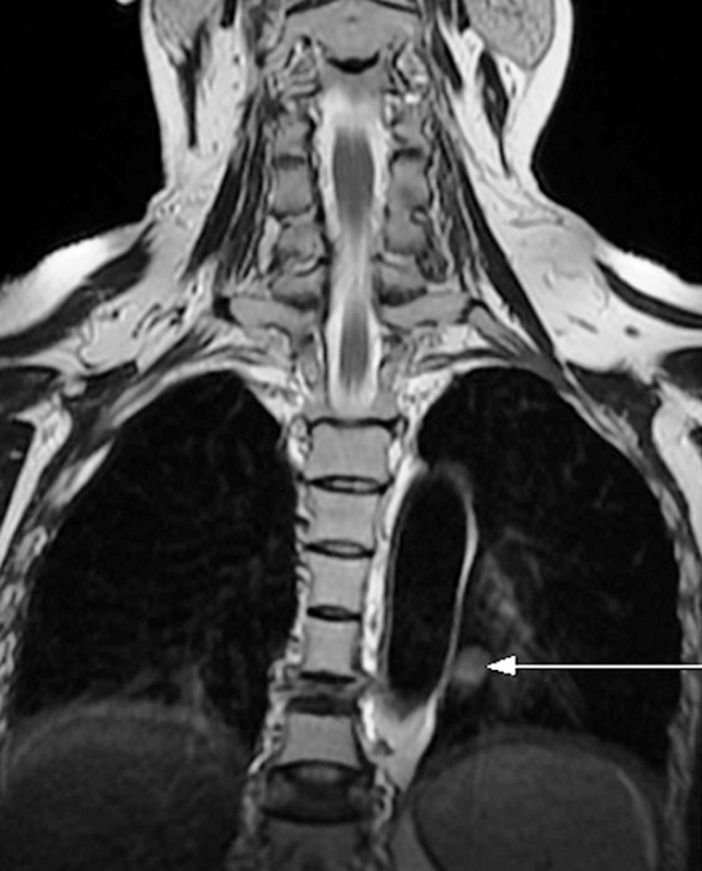
coronal chest MRI T2-weighted revealed one nodular defined lesion in the left lung (arrow)

**Figure 5:**
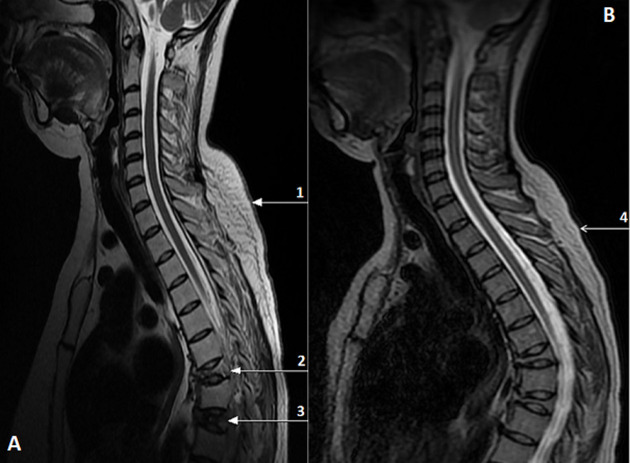
axial chest MRI T2-weighted before (A) and one year after surgery (B) reveled buffalo neck (arrow 1 and 4) and vertebral compression fractures of D7 and D9 (arrow 2 and 3)

A 31-year-old woman who consulted several times for dorsal spinal pain one year before her admission to our department for suspected CS. She accused progressive asthenia, hair loss, amenorrhea, weight gain (12 kg), severe anxiety, insomnia and depressive symptoms. The notion of taking exogenous steroids was unremarkable. In her physical examination there was non hypertention, we revealed melanodermia and Cushingoid features: increased fat around the face, neck, nape (buffalo cervix) and supraclavicular region, facial acne, (Figure 1A) and a purple striae more pronounced over her flanks and thighs (Figure 2A, Figure 3A). Following psychiatric evaluation, the patient was diagnosed with severe depression, suicidal ideation, and panic attacks. Nonspecific biology showed chronic hyperglycemia (FPG: 1.80, A1C: 7,9%), hypercholesterolemia (TC: 2.98 g/L, HDL- C: 0.69 g/L, LDL - C: 1.92 g/L, and triglycerides 1.91 g/L), hypokalemia (3.0 mmol/L), polymorphonuclear leukocytosis (WBC: 11,110/mm^3^and PMNs: 8720/mm^3^), an increase in bone turnover with increase serum alkaline phosphatase (ALP: 252 IU/L). The specific investigations were in favor of CS, the nycthemeral cortisol cycle was broken with a detectable cortisol at 23h00 (26 µg/dL), the 24h urinary free cortisol (FLU) on two consecutive days were increased (1436 µg/day and 1342 µg/day (RR < 176 µg/day)). Seen melanoderma an ACTH-dependent CS are suspected and confirmed by increased plasmatic ACTH (247 ng/L (RR: 10-50 ng/L)). The laboratory test results are summarized in Table 1. Given the high prevalence of cushing's disease, even in the absence of suppression of serum cortisol and FLU in overnight, high-dose dexamethasone suppression tests, pituitary MRI was performed and there was no identifiable tumor. It was an indication to carry out a bilateral inferior petrosal sinus sampling, but it was unavailable. Chest, abdomen, and pelvis CT scan reviewed to localize the source of ectopic cushings, revealed one nodular defined lesion (12mm) in the left lung and confirmed by MRI (Figure 4), normal adrenal glands (no signs of hyperplasia, enlargement, or nodules) and no other suspicious lesions. On the metaboic level, in addition to secondary diabetes and secondary mixed hyperlipidemia, the Bone assessment was in favor of osteporosis, Magnetic resonance imaging (MRI) shows osteoporotic vertebral compression fractures which explains dorsal spinal pain (Figure 5). The patient received a ketoconazole titration with a better reponse after two weeks on 600 mg per day in two takes (152 µg/day) without improvement in the nycthemeral cortisol cycle (high midnight cortisol). The patient underwent thoracic surgery with nodular resection by uniportal video-assisted thoracoscopic. The histology revealed a bronchial typical carcinoid tumor with positive immunostainings for for corticotropin, chromogranin A and synaptophysin. After surgical removal, we noted a complete response and the patient has adrenal insufficiency over two years (Table 1, Figure 1B, Figure 2B, Figure 3B).

**Table 1:** pertinent laboratory findings

Investigations	At admission	After Ketoconazole	After surgery	Reference range
**Non specific**				
White blood cells (k/L)	11,110	9,300	7,210	4,200 - 10,000
PMNs (k/L)	8,720	6,540	4,250	1,500 - 5,500
Potassium (mmol/L)	3	3.8	4.2	3.5 - 5.2
Bicarbonate (mmol/L)	34	27	23	20 - 30
FPG (mg/dL)	180	114	83	75 - 107
A1C (%)	7.9	-	5.7	<5.7
Total cholesterol (g/L)	2.98	1.95	2.07	1.35 - 2.07
Triglyceride (g/L)	1.91	1.62	1.35	0.22 - 1.60
Alkaline phosphatase (IU/L)	252	-	116	40 - 129
**Specific**				
Cortisol 8 AM	48.1	8.3	3.5	4.5 - 24.0
Midnight cortisol (g/dL)	26	11	0.6	<1.8
FLU (g/day)	1436	152	0.3	4.3 - 176.0
ACTH (ng/L)	247	-	12	10 - 50
Cortisol after DXM high dose	42	-	-	-
FLU after DXM high dose	1043	-	-	-

## Discussion

The incidence and the prevalence of ectopic ACTH syndrome (EAS) are imprecise because of the rarity of the disease and the underreporting of cases [2,3]. Based on the existing literature, the prevalence of EAS is between 9 and 18% of cases of ACTH-dependent CS [3]. This is a disease mainly occurring in adults and pediatric cases are exceptional [1]. Despite efficient diagnostic tests (biological, radiological and functional), EAS frequently present a major problem of positive and etiological diagnosis [2]. Indeed, when CS is suspected, the diagnosis involves three steps: confirmation of hypercortisolism, differentiation between ACTH-independent and ACTH-dependent CS, and finally differentiation between pituitary and EAS sources of ACTH. Regarding the first step, in the case of severe hypercorticism you just have to think about it, which is not obvious given non-specificity of the symptoms, especially at the start of the disease, this explains delayed diagnosis [3]. In our case, the delay between the onset of the first symptoms (dorsal spinal pain) and the diagnosis was 12 months, it was the appearance of other symptoms of hypercatabolism such as skin signs that made one think of CS and prompted a specialist consultation. To confirm the diagnosis of CS, international guidelines suggest two of four investigations: two UFC, two measures of night salivary cortisol, overnight dexamethasone suppression test (ODST) or low-dose dexamethasone suppression test [3,4]. In our context we practice 2UFC and an ODST like most centers. If two tests are positive, the diagnosis of CS is certain. The confirmation in our case was easy because of severe hypercortisolism. In the second step, clinically the presence of melanoderma was a sign in favor of a dependent ACTH CS, the ACTH dosage confirms it if it exceeds 20ng/L. In the third step, ACTH hypersecretion is more frequently of pituitary origin (Cushing's disease) or of an extrahypophysial origin in EAS or an exceptional ectopic secretion of CRH.

The differentiation between a dependent and non-dependent ACTH CS is very easy (step 2), but the differential diagnosis between a Cushing's disease and EAS presents a real challenge. It is the same for identifying the cause of EAS [2,4]. Given the severity of symptoms, normal pituitary imaging and persistence of cortisol hypersecretion after high-dose dexamethasone suppression tests, EAS was very likely. The best approach to localise the source of EAS is high-resolution imaging and it did identify a pulmonary lesion in our case. Based on the World Health Organization classification, they are two types of bronchopulmonary carcinoid tumors, typical carcinoids, which are low-grade tumors with a low mitotic rate (four times more frequent) and atypical carcinoids, which are intermediate-grade tumors with a higher mitotic rate and/or necrosis [5]. Typical carcinoids tumors can be aggressive when associated with CS as in this case [6,7]. The ideal treatment is radical excision of the tumor; it allows remission in more than 83% of cases when the lesion is unique. Medical treatment has a central role pending identification of the etiology of EAS and in preparation for surgery [3]; our case illustrates the rapid efficacy and good tolerance of ketoconazole in preparation for surgery. Survival of patients with EAS depends on the source of the primary tumour and patients with bronchial carcinoids have the best prognosis.

## Conclusion

We must remember that a tumor of benign morphology can cause malignant manifestations linked to the hormonal storm. This is the case for bronchopulmonary typical carcinoid tumor with hypercorticism which causes damage on the general, psychological and metabolic levels. Multidisciplinary approach is required for both diagnostic work-up and therapeutic management.
